# Editorial: Physiology and Clinical Potential of Eccentric Exercise

**DOI:** 10.3389/fphys.2017.00891

**Published:** 2017-11-03

**Authors:** Martino V. Franchi, Kyle W. Mitchell, Hans Hoppeler, Marco V. Narici

**Affiliations:** ^1^Laboratory for Muscle Plasticity, Department of Orthopedics, Balgrist University Hospital, University of Zurich, Zurich, Switzerland; ^2^MRC-ARUK Centre of Musculoskeletal Ageing Research, University of Nottingham, Derby, United Kingdom; ^3^Department of Anatomy, University of Bern, Bern, Switzerland; ^4^Department of Biomedical Science, Institute of Physiology, University of Padua, Padua, Italy

**Keywords:** eccentric exercise, eccentric contractions, eccentric training, rehabilitation, resistance training, mechanotransduction, muscle remodeling

“The power of locomotion is that which contracts and relaxes the muscles whereby the members and joints are moved, extended, or flexed.” This is how the Persian physician Avicenna described the crucial role of muscle contraction in human movement, reported in his masterpiece *The Canon of Medicine* (eleventh century). The ability of moving from place to place is indeed fundamental for survival: it represents a distinctive manifestation of life and evolution (Hoppeler and Herzog, 2014). Skeletal muscle allows movement through contraction and force generation: the muscle shortens in order to exert force, which is then transmitted to tendons and successively onto bones, resulting in joint movement. However, locomotion, as many other movement patterns, is a combination of distinct muscle actions: while during shortening (concentric, CON) contractions the muscle indeed shortens, lengthening (eccentric, ECC) actions allow the dissipation of mechanical energy during body deceleration (Konow and Roberts, 2015).

Thus, lengthening and shortening muscle actions coexist in many of our daily movements. Resistance training (RT) represents a widely accepted strategy of muscle conditioning that normally involves both CON and ECC. As these two types of contraction completely differ from one another in terms of mechanisms of force generation, maximum force produced and energy cost, some authors have suggested that ECC and CON would provide different stimuli for distinct muscular and functional adaptations (Roig et al., 2009). ECC actions can generate greater forces than isometric and concentric ones for any given contraction velocity (Katz, 1939). Interestingly, the metabolic cost required for ECC contractions is approximately a quarter of the one required for a CON one of the similar magnitude (Lindstedt et al., 2001). Thus, ECC RT may be particularly suitable for training individuals with medical conditions associated to muscle wasting and reduction in muscle strength, mobility, and aerobic capacity, since it provides a strong mechanical stress at a lower metabolic cost (Lastayo et al., 1999).

Despite the mentioned advantages, the use of ECC in clinical scenarios and aging has often been object of contrasting opinions, mainly because associated with exercise induced muscle damage (McHugh, 2003). However, moderate/low ECC loading has been suggested as a strategy to overcome this problem, as when monitored and progressively ramped, it effectively promotes muscle mass and strength gains without inducing muscle soreness and damage, both in lower and upper limbs (LaStayo et al., 2014; Elmer et al., 2017). Up to date, research has mainly focused on the acute and chronic effects of high-load ECC only (resistance-exercised based) while the potential of moderate/low load regimes seems to have been virtually neglected.

Through a mix of reviews, opinions and original research articles, the aim of the presen Research Topic was, on one hand, to better clarify the main physiological, functional, morphological and molecular features of ECC contractions as well as the responses of human skeletal muscle (and tendons) to ECC RT. On the other hand, we wanted to explore the feasibility of using ECC loading modalities in specific populations.

Hoppeler's review lays the groundwork of the Research Topic, discussing the main physiological features and usefulness of eccentric cycling in cardiac rehabilitation, sarcopenia, metabolic, and neurological conditions. Hoppeler makes a clear case for the application of moderate load ECC RT in cardiovascular and orthopedic rehabilitation, as well in injury prevention.

Yao et al. move the attention of the topic to the brain level, providing novel insights via fMRI analyses on the functional connectivity between the primary motor cortex and other areas during voluntary eccentric and concentric contraction.

While Nagamori et al. discuss the implications for rehabilitation purposes of bilateral alterations in leg dexterity after ECC contractions, a review article (Hessel et al.) from Professor Nishikawa's lab takes us back to fundamental muscle physiology, delineating the current understanding of muscle mechanics in ECC, focusing on titin and its proposed role in force generation.

Rindom and Vissing's mini review illustrates the beauty and complexity of molecular pathways linked to the transduction of mechanical stimuli into muscle protein accretion, discussing the implications of such cellular signaling with distinct muscle contractions.

The hypothesis and theory by Harris-Love et al. leads the way to the more “clinical” part of the topic, proposing a novel periodization model for prescription of ECC RT in outpatient rehabilitation setting. Tesch et al. illustrate the feasibility of Iso-inertial eccentric overload RT in order to counteract muscle disuse and aging, as well as deconditioning due to spaceflight, due to its applicability in rehabilitation settings. Instead, Maganaris et al. focus on human tendons and on their regional adaptations/mal-adaptations to chronic mechanical loading and tendinopathy, discussing the effectiveness of loading/rehabilitation protocols on such “topography” of responses.

The works by MacMillan et al. and Chen et al. focus, respectively, on the mitochondrial adaptations to ECC RT compared to CON RT on COPD patients and the superior effect of ECC RT (i.e., vs. CON RT) on insulin sensitivity and lipid profiles. In addition, Professor Fluck and colleagues present the cardiovascular and muscle-functional responses to work matched ECC vs. CON RT performed on a novel soft robot device which allow to prescribe rehabilitation programs tailored to the patient's functional and pathological characteristics.

Mitchell et al. wrapping up this series of articles on clinical applications of ECC RT, advocate the potential use of ECC in critical illnesses where the maintenance of muscle mass acquire major importance. In conclusion of the topic, while Hicks et al. nicely correlate muscle-tendon unit properties to the insurgence of exercise-induced muscle damage, the extensive review by Franchi et al. tries to illustrate the morphological and molecular remodeling of skeletal muscle in response to ECC and CON loading paradigms.

In conclusion, the 14 articles which constitute this research topic extensively present how ECC contractions distinguish themselves from concentric, and how could be particularly suitable for clinical scenarios. We hope that the different sides touched by the topic, from fundamental muscle and tendon physiology to motor control and brain activity, have provided a clearer idea of what we know about ECC so far, hopefully helping to move this research field forward.

## Author contributions

MF conceived the manuscript. MF, KM, HH, and MN contributed to the final editing of the manuscript.

### Conflict of interest statement

The authors declare that the research was conducted in the absence of any commercial or financial relationships that could be construed as a potential conflict of interest.
